# Exosomes-loaded with traditional Chinese medicine constituents: a novel therapeutic avenue for aplastic anemia

**DOI:** 10.1186/s13020-026-01330-2

**Published:** 2026-01-26

**Authors:** Guo-Kai Zhang, Fang Zhou

**Affiliations:** 1 The First Clinical Medical School, Shandong University of Traditional Chinese Medicine, Jinan, China; 2 Department of Hematology, the 960th Hospital of the People’s Liberation Army Joint Logistics Support Force, Jinan, China

**Keywords:** Exosomes, Traditional Chinese medicine constituents, Aplastic anemia, Drug delivery system

## Abstract

Aplastic anemia (AA) is a life-threatening blood disorder characterized by bone marrow failure and pancytopenia. Treatments such as immunosuppressive therapy (IST) and allogeneic hematopoietic stem cell transplantation (allo-HSCT) have limitations, including poor long-term remission and donor shortages. Exosomes, nanosized extracellular vesicles, are promising drug delivery systems (DDS) due to their biocompatibility, targeted delivery, and capacity to penetrate biological barriers. Traditional Chinese medicine (TCM) regulates hematopoiesis and immunity but faces challenges with delivery. This review discusses exosomes loaded with TCM constituents (Exo-TCM) for AA, summarizing AA pathogenesis, exosome features, TCM potential, Exo-TCM preparation, and preclinical efficacy, and translational challenges, highlighting Exo-TCM as a novel therapeutic approach for AA.

**Graphical Abstract:**

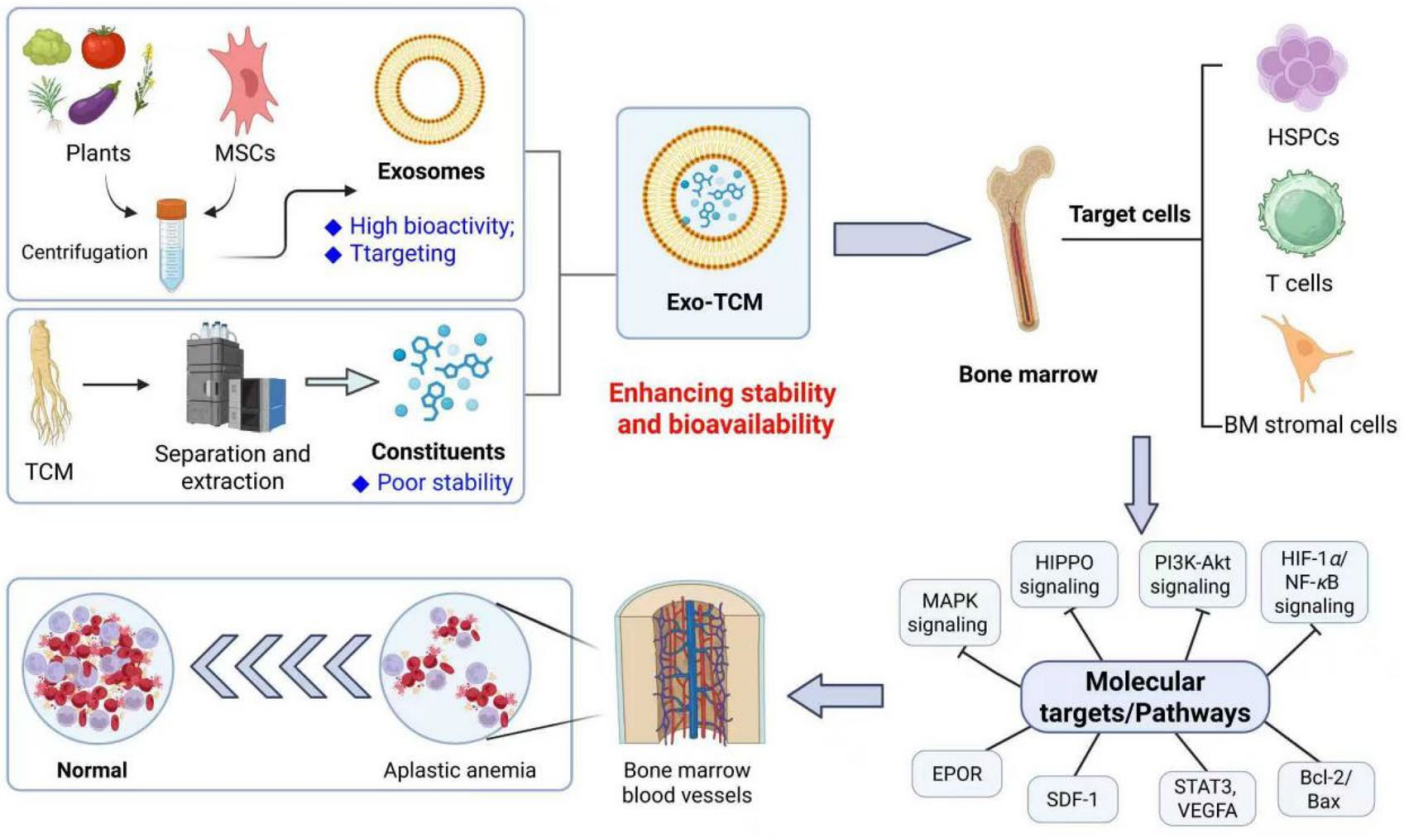

## Introduction

Aplastic anemia (AA) is a rare but life-threatening bone marrow (BM) failure syndrome characterized by pancytopenia and hypocellular BM without abnormal infiltration or fibrosis [1–3]. The pathogenesis of AA remains incompletely understood, but current evidence points to immune-mediated destruction of hematopoietic stem cells as the predominant mechanism [4–6]. Autoimmune abnormalities, particularly imbalanced Th17/Treg ratios and dysregulated MAIT cell frequencies in the BM, have been implicated in the disease progression [7, 8]. Additionally, alterations in the hematopoietic microenvironment, including aberrant expression of genes like VEGFA, ANGPT1, and SDF-1 in mesenchymal stem cells, may contribute to the pathogenesis [9]. While environmental triggers (e.g., viral infections) and genetic predispositions are suspected initiating factors, the exact etiology remains idiopathic in most cases [10]. Clinically, AA presents significant challenges in both diagnosis and management. The disease manifests with symptoms related to pancytopenia, including infections, bleeding, and fatigue [11]. Severe AA (SAA) carries high mortality if untreated [12, 13], necessitating early intervention with either IST using anti-thymocyte globulin combined with eltrombopag, or hematopoietic stem cell transplantation (HSCT) [14]. However, treatment complications such as sepsis during transplant waiting periods [15] and high malnutrition rates (51.3% overall) further complicate management [16]. Emerging metabolomic studies suggest potential biomarkers for SAA [17], while novel therapeutic approaches targeting microRNA regulation show promise in correcting immune imbalances [8]. The heterogeneity of AA, including rare presentations with hypercellular marrow [18] and associations with conditions like generalized morphea [15], underscores the need for continued research to elucidate disease mechanisms and optimize therapeutic strategies.

Exosomes, have garnered significant attention as promising drug delivery carriers due to their natural origin, high biocompatibility, low immunogenicity, and innate ability to overcome biological barriers [19–21]. These nanoscale vesicles play pivotal roles in intercellular communication and material transfer, making them ideal for targeted drug delivery applications. Recent advances highlight their potential in cancer therapy, where exosome-mediated delivery of chemotherapeutics, nucleic acids, and proteins demonstrates enhanced therapeutic efficacy with reduced off-target effects compared to conventional methods [22–24]. The development of exosome-based drug carriers involves critical steps such as isolation, characterization, and drug loading. Techniques like sonication and surface modification are employed to optimize cargo encapsulation and tumor-targeting capabilities [25, 26]. Mesenchymal stem cell (MSC)-derived exosomes are particularly notable for their therapeutic potential, offering advantages like natural tropism to disease sites and stimulation of anti-cancer immune responses [27]. However, challenges persist, including low yield, heterogeneity, and limited drug-loading efficiency, which hinder clinical translation [28]. Innovative solutions, such as utilizing milk-derived exosomes or plant exosome-like nanoparticles, are being explored to address scalability and source limitations [29, 30]. Future research directions emphasize engineering exosomes with biomaterials to enhance functionality and homogeneity, paving the way for their application in regenerative medicine and precision therapy [31, 32]. Despite existing hurdles, exosomes represent a transformative platform in nanomedicine, bridging the gap between laboratory research and clinical implementation [33].

TCM constituents have demonstrated significant therapeutic potential in the treatment of hematological diseases through diverse mechanisms. TCM monomers and compounds, such as those derived from Astragalus and Angelica, exhibit promising effects in ameliorating myelosuppression by modulating hematopoietic stem cells, immune factors, and critical signaling pathways like TGF-*β*/Smad [34]. These natural products are particularly valuable due to their antioxidant properties, ability to stimulate hematopoietic stem cell proliferation, and capacity to mitigate oxidative stress [35]. For anemia, TCM prescriptions such as Shengyu Decoction contain multiple bioactive compounds that address hematological dysfunction, though their pharmacodynamic basis remains under study [36]. In AA, TCMs been shown to improve patient survival as a complementary therapy [37]. The development of databases like DCABM-TCM facilitates the identification of blood-absorbed TCM constituents, enabling network pharmacology analyses to elucidate molecular mechanisms and discover bioactive compounds [38]. Despite these advances, challenges remain in quantifying constituents and clarifying absorption properties of specific herbs like *Spatholobi Caulis* [39, 40]. Overall, TCM constituents represent a rich resource for developing targeted, low-toxicity therapies for hematological diseases.

TCM constituents, despite structural complexity and poor stability, offer immunomodulatory and hematopoietic benefits, but their delivery is challenging [41, 42]. Exosomes, with high biocompatibility and targeting capacity, may serve as ideal carriers for TCM compounds, enhancing their stability and bioavailability. For instance, exosome-based DDS could ameliorate TCM's limitations while leveraging their natural therapeutic effects, such as modulating HSPC apoptosis or cytokine storms in AA. Additionally, plant-derived exosome-like nanovesicles (PELNs) from TCM herbs show promise in disease treatment and nanoparticle delivery, further supporting their integration with MSC-Exos for AA therapy [43]. Combined, Exo-TCM may synergistically restore immune homeostasis (e.g., Th17/Treg balance) and promote hematopoiesis, offering a novel strategy for AA management [44].

**Fig. 1:**
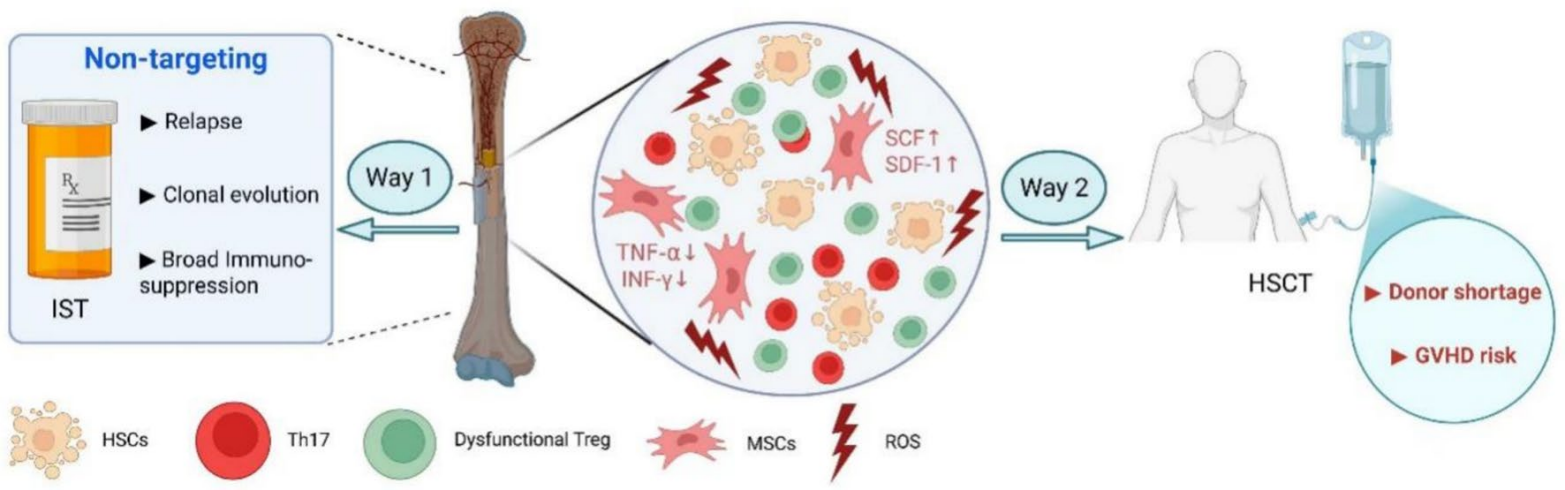
The multifactorial pathogenesis of AA and the rational for novel combinatorial therapy. In the diseased BM niche, aberrantly activated T lymphocytes (e.g., Th17 cells) initiate immune attack against hematopoietic stem/progenitor cells (HSPCs), leading to their apoptosis and depletion. Concurrently, mesenchymal stem cells and other stromal components exhibit impaired function, resulting in a deficient supportive microenvironment characterized by reduced secretion of essential hematopoietic factors (e.g., SCF, SCF-1) and increased inflammatory cytokines (e.g., TNF-*α*, IFN-*γ*). Widespread oxidative stress (ROS) further exacerbates HSPC damage and niche dysfunction. These events collectively lead to BM failure and pancytopenia. The limitations of primary current therapies are highlighted: IST acts through broad immunosuppression with risks of relapse and clonal evolution, while HSCT is constrained by donor availability and GVHD. This landscape necessitates the development of targeted, multitargeted approaches (created with BioRender.com.)

The complex and interrelated pathogenesis of AA, encompassing immune-mediated destruction of HSCs, a dysfunctional bone marrow microenvironment (BMM), and the resultant pancytopenia, alongside the limitations of current standard therapies, is schematically summarized in Fig. 1. This multifaceted pathophysiology underscores the critical need for novel therapeutic strategies that can concurrently target these multiple defective components.

## Exosomes as DDS: properties and modification

### Physicochemical properties of exosomes

Exosomes are nanoscale extracellular vesicles (30–150 nm) with a lipid bilayer membrane, secreted by nearly all cell types through the endosomal pathway [45, 46]. These vesicles exhibit unique physicochemical characteristics that underpin their biological functions and therapeutic potential. Structurally, exosomes display a typical cup-shaped morphology, as confirmed by high-resolution imaging techniques, with size variations observed across cell types (e.g., 82.6–118.3 nm in PBS- vs virus-stimulated exosomes) [47]. Their membrane composition reflects the parental cell's lipid and protein profile, containing characteristic markers (CD81, CD9, CD63) while carrying diverse cargoes including proteins, nucleic acids (miRNA, mRNA, DNA), and lipids [48, 49].

Key physical properties include: (1) Density close to body fluids (1.13–1.19 g/mL), posing challenges for isolation [50]; (2) Negative surface charge that varies with pathological conditions, neuroblastoma exosomes show increased surface charge when exposed to A*β* oligomers [51]; (3) Mechanical properties measurable via atomic force microscopy, where HepG2-derived exosomes demonstrated quantifiable stiffness [52]. These parameters influence exosome behavior, including their stability, cellular uptake efficiency, and biodistribution [53].

Chemically, exosomes exhibit remarkable biocompatibility, low immunogenicity, and an inherent ability to cross biological barriers [54]. Their surface composition enables selective targeting, while the lipid bilayer protects labile cargo during circulation [55]. Plant-derived exosomes display broader size distributions (50–200 nm) and distinct physicochemical profiles compared to animal counterparts. The vesicles' lipophilicity and molecular weight significantly affect drug loading efficiency, a critical parameter for therapeutic applications [56]. These physicochemical properties collectively facilitate exosomes' roles in intercellular communication and make them promising candidates for drug delivery, diagnostic biomarkers, and regenerative medicine [57]. However, their heterogeneity necessitates careful characterization when developing isolation and engineering strategies [58, 59].

### Exosome source selection and functional heterogeneity for AA therapy

The therapeutic efficacy of exosomes is intrinsically linked to their cellular origin, as their cargo (proteins, lipids, nucleic acids) and surface composition are a "fingerprint" of the parent cell. Therefore, selecting an appropriate exosome source is a critical first step in designing effective Exo-TCM for AA, which requires simultaneous targeting of HSPCs, immune cells, and the BM microenvironment.

#### Mesenchymal stem cell-derived exosomes (MSC-Exos)

MSC-Exos have emerged as a promising therapeutic strategy for AA due to their unique immunomodulatory and regenerative properties. Studies have demonstrated that MSC-Exos can partially substitute the immunoregulatory functions of MSCs, addressing the dysfunction observed in AA patients' MSCs [60]. These exosomes play a crucial role in modulating immune responses by regulating CD3 + T cells, which are implicated in the immunologic dissonance characteristic of AA. Compared to whole MSC transplantation, MSC-Exos offer several advantages, including reduced risk of immune rejection and ethical concerns, as well as enhanced safety profile [61]. The therapeutic efficacy of MSC-Exos is attributed to their cargo of bioactive molecules, such as proteins, miRNAs, and lipids, which mediate cytoprotection, anti-apoptotic effects, and tissue regeneration [62, 63]. Preclinical evidence suggests that MSC-Exos can promote hematopoietic recovery and modulate inflammatory responses in AA models [64]. Additionally, their cell-free nature allows for targeted delivery and sustained release, overcoming limitations associated with poor homing efficiency and low survival rates of transplanted MSCs [65]. While challenges remain in optimizing isolation and delivery methods, MSC-Exos represent a novel and potentially transformative approach for AA treatment, offering advantages over conventional therapies by addressing both the hematopoietic and immune dysregulation components of the disease.

#### HSPC-derived exosomes

HSPC-derived exosomes exhibit significant therapeutic potential in AA These nanovesicles inherit bioactive molecules from parental cells, enabling them to modulate immune responses and promote hematopoietic recovery without eliciting GVHD risks associated with allogeneic transplantation [66, 67]. Studies demonstrate that exosomes from MSCs partially replicate MSC immunoregulatory functions, addressing the immunologic dissonance observed in AA patients where dysfunctional AA-MSC-derived exosomes contribute to disease progression [60]. Notably, exosomes can deliver trophic factors and miRNAs to residual HSPCs, stimulating their proliferation and differentiation—a mechanism analogous to Eltrombopag's trilineage hematopoietic expansion effects [68, 69]. Their ability to cross biological barriers allows targeted delivery of immunomodulatory cargo (e.g., TGF-*β*, IL-10) to suppress pathogenic T-cell responses driving HSPC destruction [70, 71]. Compared to conventional immunosuppressive therapy (IST), exosomes offer reduced toxicity while potentially overcoming limitations of IST non-responders with severely depleted HSPC pools [72]. For hepatitis-associated AA, exosome therapy may circumvent liver toxicity risks posed by IST or transplantation [73]. Furthermore, exosomes from HLA-engineered HSPCs could mitigate immune escape mechanisms seen in AA patients with somatic HLA loss [74]. The applicability extends to pediatric and elderly populations ineligible for hematopoietic stem cell transplantation (HSCT), as exosomes lack donor-matching requirements and conditioning regimen toxicities [75]. Emerging evidence also suggests their role in repairing BM stromal defects secondary to hematopoietic failure [76]. While clinical translation requires standardization of isolation protocols and dosing, HSPC-derived exosomes represent a promising cell-free therapeutic paradigm for AA by integrating immunomodulation, hematopoietic support, and microenvironmental repair [77, 78].

#### Immune cells derived exosome

Exosomes derived from immune cells such as dendritic cells (DCs) and regulatory T cells (Tregs) exhibit significant therapeutic potential in immune-mediated AA by modulating the dysregulated immune microenvironment. DC-derived exosomes (DC-EXOs) possess the unique ability to be engineered with immunoregulatory molecules, allowing for targeted immune modulation. These exosomes can suppress pathogenic T-cell responses, particularly cytotoxic Th1 cells that drive HSPC destruction in AA [60]. Notably, DC-EXOs have demonstrated favorable safety profiles in clinical trials for inflammatory diseases, suggesting their translational applicability in AA. Treg-derived exosomes may compensate for the impaired immunosuppressive function of mesenchymal stem cell (MSC)-derived exosomes observed in AA patients, potentially restoring immune homeostasis [79]. The therapeutic advantage lies in their ability to recapitulate the immune-regulatory functions of parent cells while avoiding cellular therapy risks. Exosomes from immune cells can disrupt the pathological immune network involving T-, B-, and myeloid cells that contributes to AA pathogenesis [80]. Particularly, they may counteract the aberrant activation of STAT1 signaling observed in AA patients [81] and mitigate the effects of proinflammatory cytokines like IFN-*γ* that sustain the autoimmune attack [82]. Their nanoscale size enables efficient biodistribution to the BM niche, where they can directly interact with resident immune cells and HSPCs [83]. Importantly, immune cell-derived exosomes may overcome limitations of current immunosuppressive therapies by targeting multiple pathogenic mechanisms simultaneously, including T-cell activation, myeloid-derived suppressor cell (MDSC) dysfunction, and antigen presentation abnormalities [84, 85]. The ability to combine exosome therapy with existing regimens like cyclosporine could enhance treatment efficacy while reducing toxicity [86]. These features position immune cell-derived exosomes as promising candidates for developing precision immunomodulatory strategies in AA.

#### PELNs

PELNs share structural similarities with mammalian exosomes, containing bioactive components such as lipids, proteins, microRNAs, and plant-derived metabolites that enable cross-kingdom communication with human cells [87, 88]. Compared to MSC-derived exosomes, which exhibit dysfunction in AA patients [60], PELNs offer several advantages, including low immunogenicity, high bioavailability, and scalable production from diverse plant sources [89, 90]. The lipid bilayer structure of PELNs protects their cargo and facilitates efficient cellular uptake through multiple endocytic pathways [91], allowing delivery of immunoregulatory molecules that may restore hematopoietic function. Particularly relevant for AA treatment, PELNs have demonstrated potent immunomodulatory effects in inflammatory conditions through mechanisms involving microRNA-mediated regulation of immune cells and cytokine networks [92, 93]. Their ability to modulate the intestinal microecological balance suggests potential for systemic immune regulation [94, 95], which could address the immunologic dissonance characteristic of AA pathogenesis. Furthermore, PELNs exhibit superior stability and lower production costs compared to mammalian-derived exosomes [96], making them more feasible for clinical translation. The presence of plant-specific bioactive compounds in PELNs may provide additional therapeutic benefits, such as anti-inflammatory and tissue-protective effects [97, 98]. While direct evidence in AA models remains limited, the established capacity of PELNs to regulate immune responses and promote tissue repair [99, 100] positions them as a novel, naturally-derived therapeutic strategy worthy of further investigation for this hematologic disorder.

The choice of exosome source should align with the primary therapeutic goal of the Exo-TCM formulation. For instance, MSC-Exos are suited for comprehensive niche repair and immunomodulation, while HSPC-Exos might be optimal for direct hematopoietic stimulation. Future research should compare the efficacy of Exo-TCMs derived from different sources in AA models and explore hybrid strategies, such as using engineered cells that overexpress specific therapeutic factors to produce "super-exosomes".

#### Surface modification of exosomes for targeted delivery

Surface modification of exosomes has emerged as a pivotal strategy to enhance their targeting capabilities for drug delivery applications. Exosomes, naturally derived nanovesicles with inherent biocompatibility and low immunogenicity, possess surface biochemistry reflective of their parent cells, yet require engineering to achieve tissue-specific delivery [101, 102]. Current modification approaches encompass genetic, chemical, and physical methods to functionalize exosome surfaces with targeting moieties such as aptamers, antibodies, peptides, and polymers [103, 104]. Genetic engineering enables stable expression of targeting ligands (e.g., peptides or antibody fragments) on exosomal membranes, while chemical conjugation methods like cholesterol-modified DNA tethers allow precise attachment of functional polymers to modulate pharmacokinetics [105, 106]. Notably, phage display technology has been employed to identify targeting peptides for pulmonary diseases, demonstrating the potential of peptide-modified exosomes in organ-specific delivery [20]. Surface-engineered exosomes exhibit enhanced therapeutic efficacy in diverse applications, including neurodegenerative disease treatment through improved blood–brain barrier (BBB) penetration [107], and cartilage-targeted delivery of Nrf2 for antioxidant therapy in intervertebral disc degeneration [108]. The integration of stimuli-responsive components (e.g., pH-sensitive polymers or enzyme-cleavable ligands) further enables smart exosomes to overcome biological barriers like the tumor microenvironment [109]. Despite progress, challenges persist in clinical translation, including standardization of modification techniques and scalability of production [110, 111]. Future directions emphasize combinatorial strategies (e.g., dual genetic-chemical modifications) to optimize targeting precision while maintaining exosome stability. These advances position surface-modified exosomes as transformative tools for precision medicine, particularly in oncology and regenerative therapies [26, 112].

In AA, the key target cells are HSPCs (to restore hematopoiesis), T cells (to regulate immunity), and BM stromal cells (to repair the microenvironment).

**Fig. 2:**
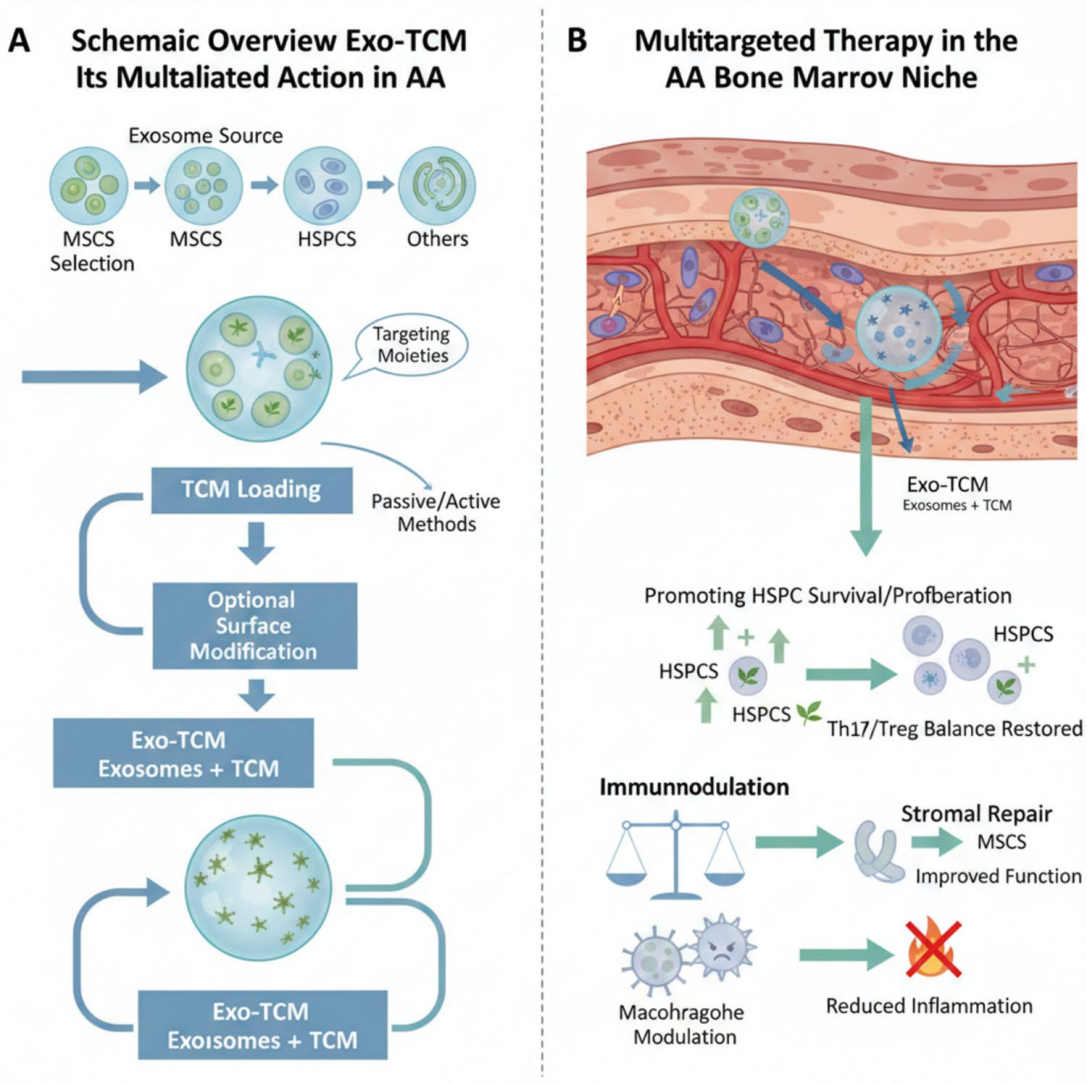
Schematic overview of Exo-TCM engineering and its multifaceted action in AA. **A** The Exo-TCM preparation pipeline, encompassing selection of exosome source, loading of TCM constituents via passive/active methods, and optional surface modification for targeting. **B** Upon systemic delivery, Exo-TCM exerts coordinated therapeutic effects within the diseased bone marrow niche: promoting HSPC survival/proliferation, restoring immune homeostasis (e.g., Th17/Treg balance), modulating macrophage polarization, and repairing stromal cell function to mitigate inflammation and niche dysfunction (created with BioRender.com.)

The selection of an appropriate exosome source is the first critical step in designing Exo-TCM. Following this selection, the subsequent engineering pipeline, including drug loading and optional modification-is essential to create a functional therapeutic agent. The overall preparation workflow is conceptually outlined in Fig. 2A.

### Biocompatibility and safety of exosomes

Exosomes' innate properties include low immunogenicity, high stability, and the ability to traverse biological barriers, which collectively distinguish them from synthetic nanoparticles [113–116]. Derived from diverse cell types, exosomes mimic native cellular membranes, reducing adverse immune reactions and enhancing biodistribution [24, 116]. Studies highlight their utility in drug delivery, where their biocompatibility permits efficient cargo transport (e.g., chemotherapeutics, nucleic acids) with minimal toxicity [117]. For instance, plant-derived exosomes demonstrate safety and anticancer potential due to their cell-free nature and nanoscale structure, while mesenchymal stem cell (MSC)-exosomes leverage biocompatibility for targeted therapy with reduced off-target effects [118].

Safety evaluations in lung diseases and multiple myeloma (MM) further underscore exosomes' feasibility as cell-free therapeutics [119, 120]. Their biodegradability and surface modifiability (e.g., targeting ligands) enhance specificity and minimize systemic toxicity [111, 121]. However, challenges such as heterogeneous cargo, low yield, and standardization hurdles persist [33]. Engineered exosomes, through surface functionalization or hybrid synthesis, aim to address these limitations while preserving biocompatibility [122]. Overall, exosomes' dual advantages of safety and biocompatibility position them as transformative tools in regenerative medicine, diagnostics, and targeted therapy [123–125].

## TCM constituents with potential in AA treatment

### Active components of TCM and their mechanisms

#### Ginsenoside Rg1

Ginsenoside Rg1 (Table 1), derived from *Panax ginseng*, exhibits hematopoietic protection in AA models. In myelosuppressive mice, Rg1 significantly ameliorated BM hypocellularity and peripheral blood cytopenia by modulating oxidative stress and apoptosis pathways. It upregulated hematopoietic stem cell (HSC) proliferation markers while suppressing pro-inflammatory cytokines (e.g., TNF-*α*, IL-6), suggesting dual roles in hematopoiesis promotion and immune regulation [126, 127].

**Table 1:** Active constituents from TCM and their mechanisms of action in the treatment of AA

TCM constituents	Source	Proposed mechanisms of action	Molecular targets/Pathways	Experimental model	Refs.
Ginsenoside Rg1	*Panax ginseng*	Promotes proliferation and inhibits apoptosis of HSPCs; reduces pro-inflammatory cytokines	MAPK signaling pathway; ROS↓, TNF-*α*↓, IL-6↓	Cyclophosphamide-induced myelosuppression in mice	[126, 127]
APS	*Astragalus membranaceus*	Enhances HSPCs self-renewal and reduces apoptosis; improves BMM	Inhibits Hippo pathway effector YAP phosphorylation; modulates Bcl-2/Bax ratio	AA mouse model	[128]
SCC	Semi-synthetic derivative of chlorophyll	Promotes erythroid and myeloid differentiation; attenuates oxidative damage in marrow stromal cells	Upregulates EPOR expression; antioxidant effects	AA rat model	[129]
Fuzi-derived alkaloids	*Aconitum carmichaelii*	Promotes HSC survival under stress; potentially modulates angiogenesis and immunity	Targets PI3K-Akt and HIF-1 signaling pathways; binds to STAT3, VEGFA (in silico)	Network pharmacology and in vitro studies	[130]
RGP	*Rehmannia glutinosa*	Enhances integrity of the BMM; promotes HSC homing	Upregulates SDF-1; modulates HIF-1*α*/NF-*κ*B signaling	Busulfan-induced AA mice	[132]

#### Astragalus polysaccharide (APS)

APS (Table 1), isolated from *Astragalus membranaceus*, targets the Hippo pathway-a critical regulator of HSC self-renewal. In AA mice, APS restored blood counts and marrow cellularity by inhibiting Hippo effector YAP phosphorylation, thereby promoting HSC proliferation. Additionally, APS reduced apoptosis in CD34 + cells via Bcl-2/Bax pathway modulation, highlighting its potential to counteract marrow failure [128].

#### Sodium copper chlorophyllin (SCC)

SCC (Table 1), a semi-synthetic derivative of chlorophyll, demonstrated efficacy in AA rats by enhancing erythroid and myeloid lineage differentiation. It attenuated oxidative damage in marrow stromal cells and upregulated erythropoietin receptor (EPOR) expression, suggesting a role in mitigating oxidative stress-mediated HSC suppression [129].

#### Fuzi-derived alkaloids

*Aconitum carmichaelii* (Fuzi, Table 1) contains bioactive alkaloids (e.g., magnoflorine, scoulerine) identified via network pharmacology. These compounds target PI3K-Akt and HIF-1 signaling pathways, which are crucial for HSC survival under hypoxic stress. Molecular docking confirmed their binding affinity to key proteins like STAT3 and VEGFA, implicating angiogenesis and immune modulation in AA recovery [130].

#### Er-xian decoction (EXD) components

EXD, a TCM formula, contains monomers (e.g., icariin, berberine) that regulate AA-related targets (e.g., TP53, JAK2). These components modulate apoptosis and cytokine production (e.g., IL-17, IFN-*γ*), restoring Th17/Treg balance—a key imbalance in AA pathogenesis [8, 131].

#### Rehmannia glutinosa polysaccharide (RGP)

RGP (Table 1) improved hematopoietic function in busulfan-induced AA mice by enhancing BMM integrity. It increased stromal cell-derived factor-1 (SDF-1) secretion, promoting HSC homing and niche support [132].

### Mechanisms of TCM in treating AA

#### Immunomodulation and T-cell regulation

TCM formulas like EXD and “Modified Guilu Erxian Glue (MGEG)” target immune dysregulation in AA. Network pharmacology analyses reveal that EXD's active components (e.g., flavonoids, terpenoids) modulate genes such as STAT3 and NF-*κ*B, attenuating aberrant T-cell activation and restoring Th17/Treg balance [133]. Similarly, MGEG, combining “Guilu Erxian Glue” and “Danggui Buxue Tang”, tonifies kidney and blood production, suppressing pro-inflammatory cytokines (e.g., TNF-*α*, IL-6) via PI3K/Akt signaling [134]. Formononetin, a bioactive compound from “Huangqi”, specifically enhances Treg function while inhibiting Th17 differentiation, rebalancing immune homeostasis in SAA.

#### Hematopoietic microenvironment restoration

TCM interventions like RGP and APS repair the BMM. RGP reverses busulfan-induced BMM damage by upregulating VEGF and SCF, critical for HSC niche maintenance [132]. APS activates the Hippo pathway effector YAP1, promoting HSC proliferation and reducing apoptosis in AA mice [128]. Additionally, “Shenlu granule” significantly improves hemograms and TCM syndrome scores by enhancing SDF-1 secretion, facilitating HSC homing [135].

#### Mitochondrial autophagy and oxidative stress mitigation

“Modified Shisiwei Jianzhong Decoction (SJD)” ameliorates AA via mitophagy induction. Network pharmacology and animal experiments demonstrate that SJD's compounds (e.g., berberine) activate PINK1/Parkin-mediated mitophagy, clearing damaged mitochondria and reducing oxidative stress in hematopoietic cells [136]. Ginsenoside Rg1, another TCM-derived agent, exhibits myeloprotective effects by upregulating Nrf2/HO-1 antioxidant pathways, mitigating radiation-induced marrow suppression [126, 127].

#### Multi-component synergy and clinical integration

TCM's holistic approach leverages herb pairs (e.g., “*Angelicae Sinensis Radix*” in “Xin-Sheng-Hua Granule”) to synergistically improve erythropoiesis in hemolytic AA models [137]. Clinical studies highlight the efficacy of combined TCM-western regimens, such as “Lanzhou prescription” with IST, which enhances response rates in pediatric AA by reducing “heat toxin” accumulation [138]. Data mining further identifies high-frequency TCM herbs for AA, reflecting core principles of “kidney-tonifying” and “blood-nourishing” [139].

TCM treats AA through multi-target mechanisms encompassing immunomodulation, BMM repair, and mitochondrial homeostasis. Future research should validate these pathways via high-throughput omics and randomized trials to optimize TCM integration into AA therapy.

### Challenges in TCM constituent delivery

The effective delivery of TCM constituents faces significant challenges due to their inherent physicochemical and biological properties. A primary obstacle is the poor water solubility of many TCM active ingredients (e.g., terpenoids, polyphenols, alkaloids), which limits their dissolution and absorption, thereby reducing bioavailability [140–142]. Additionally, the multi-component nature of TCM formulations-comprising alkaloids, flavones, glycosides, and volatile introduces complexity in formulating stable and reproducible DDSs [143, 144]. The BBB further complicates delivery for neurological disorders like Alzheimer's disease, as most TCM constituents cannot cross this barrier unaided, diminishing therapeutic efficacy [38, 145]. Systemic delivery is also hampered by rapid metabolism in the gastrointestinal tract and liver, as well as interactions with gut microbiota, which may degrade or transform constituents before they reach systemic circulation [144, 146]. Toxicity and stability issues arise from the narrow therapeutic windows of some TCM compounds, necessitating precise dosing and targeted delivery to minimize off-target effects. Conventional formulations often fail to address these challenges, highlighting the need for advanced DDSs such as nanocarriers (e.g., liposomes, polymeric nanoparticles) to enhance solubility, prolong circulation, and enable tissue-specific targeting [147]. However, scaling up nanocarrier-based systems for clinical use remains hindered by manufacturing complexity, cost, and regulatory hurdles [147, 148].

## Exo-TCM: preparation and characterization

### Loading methods

The choice of loading method for Exo-TCM depends on the TCM constituent’s properties (solubility, stability) and the desired loading efficiency. This section compares passive and active loading methods, with a focus on their application to TCM constituents (Table 2).

**Table 2:** Loading efficiency and stability of TCM constituents by different; loading methods

Loading methods	Principle	Key parameters	Loading efficiency	Stability	Advantages	Disadvantages	Recommended
Incubation	Passive diffusion across the lipid membrane	Time (12–48 h); temperature (37 °C);	28–35	~ 15%	Simple; preserves exosome integrity and functionality	Low efficiency; suitable only for small lipophilic molecules	Hydrophobic drugs (e.g., Baicalin)
Sonication	Membrane disruption by acoustic energy followed by reassembly	Power (e.g., 200 W); duration (e.g., 3 min); pulse on/off cycle	45–55	~ 22%	Significantly improved loading efficiency	Potential damage to exosome membrane and surface proteins; increased leakage	Robust, hydrophobic compounds
Electroporation	Transient pore formation via electrical pulses	Voltage (e.g., 200–500 V); pulse length (e.g., 3–10 ms)	60–70	~ 19%	High efficiency for hydrophilic molecules and nucleic acids	Can cause drug aggregation; potential exosome fusion/aggregation	Hydrophilic drugs, nucleotides (e.g., AMP)
Freeze–thaw cycles	Membrane destabilization through ice crystal formation	Number of cycles (e.g., 3–5); Freezing temperature (-80 °C)	30–40	~ 17%	Simple equipment; no chemical solvents required	Moderate efficiency; can compromise membrane integrity	Various compound types
Saponin permeabilization	Cholesterol binding to create membrane pores	Saponin concentration (e.g., 0.1–0.5%); incubation time	40–50	~ 14%	Good efficiency with minimal impact on exosome size	Requires post-loading purification to remove detergent	Medium-sized molecules
Extrusion	Mechanical forcing through porous membranes	Pore size (e.g., 100–400 nm); number of passes	50–65	~ 20%	Homogeneous exosome size distribution; good efficiency	High shear stress may damage exosomes and cargo	Pre-formed exosomes and drug mixtures

#### Passive loading

Passive loading represents a fundamental strategy for encapsulating therapeutic agents into exosomes, leveraging their natural cargo-loading mechanisms without external manipulation. This approach exploits the inherent biocompatibility and low immunogenicity of exosomes, making them suitable for delivering complex TCM compounds, which often face challenges such as poor stability and unclear mechanisms [41, 42]. Passive loading typically involves co-incubation of exosomes with hydrophobic or small-molecule drugs, allowing diffusion across the exosomal membrane [149, 150]. Studies suggest that factors like incubation time (e.g., 48 h) and mechanical force (e.g., 1 g/cm^2^) can optimize loading efficiency without compromising exosome integrity [151]. TCM-derived ELNs further enhance passive loading due to their natural affinity for plant bioactive compounds, offering a promising platform for nanoparticle delivery [43]. However, limitations include low encapsulation efficiency for large or hydrophilic molecules, necessitating further exploration of hybrid loading techniques [152]. Future research should standardize passive loading protocols to facilitate clinical translation of TCM-exosome therapeutics.

#### Active loading

Active loading represents a pivotal strategy for enhancing the therapeutic payload of exosomes, particularly for delivering complex TCM compounds. Unlike passive loading, which relies on diffusion or incubation, active loading employs physical or chemical methods to directly encapsulate bioactive TCM ingredients into exosomes, ensuring higher efficiency and stability [22, 149]. Techniques such as electroporation, sonication, and freeze–thaw cycles are commonly utilized to facilitate the integration of TCM active components—often characterized by poor solubility or structural complexity—into exosomal vesicles [25] For instance, electroporation induces transient membrane pores, enabling the entry of TCM small molecules (e.g., ginsenosides or podophyllotoxin derivatives) into exosomes, while sonication disrupts lipid bilayers to improve drug-loading capacity [153]. The selection of active loading methods is guided by TCM-specific challenges, including the need to preserve the structural integrity of multi-component formulations (e.g., herbal extracts like Ginkgo biloba or LWDH Pill) and to overcome low natural encapsulation rates [154, 155]. Recent advances highlight the combination of active loading with exosome modification (e.g., surface ligand engineering) to achieve targeted delivery of TCM compounds, thereby addressing issues like off-target effects and rapid clearance341. Furthermore, bioinformatics tools (e.g., TCMSP, TCMID) aid in screening TCM ingredients with optimal ADME properties for active loading, ensuring compatibility with exosomal carriers [156, 157]. Despite progress, challenges persist in standardizing loading efficiency and scalability for clinical translation. Future research should optimize parameters (e.g., electric field strength for electroporation) and explore hybrid strategies (e.g., active–passive co-loading) to maximize the therapeutic potential of TCM-loaded exosomes [152].

In conclusion, both passive and active loading methods are pivotal for TCM-exosome formulations, with selection dependent on the physicochemical properties of the TCM constituents and therapeutic goals. Passive loading suits lipophilic, low-MW compounds, while active strategies are indispensable for complex TCM molecules. Future studies should standardize protocols to balance EE, exosome integrity, and scalability for clinical translation.

### Synergistic mechanisms between exosomes and TCM constituents

The integration of TCM constituents with exosomes is not merely a combination of a delivery vehicle and a payload; it represents the formation of a novel therapeutic entity with emergent properties. The interactions between exosomes and TCM constituents can be multifaceted, leading to enhanced therapeutic outcomes beyond the simple sum of their parts.

**Fig. 3:**
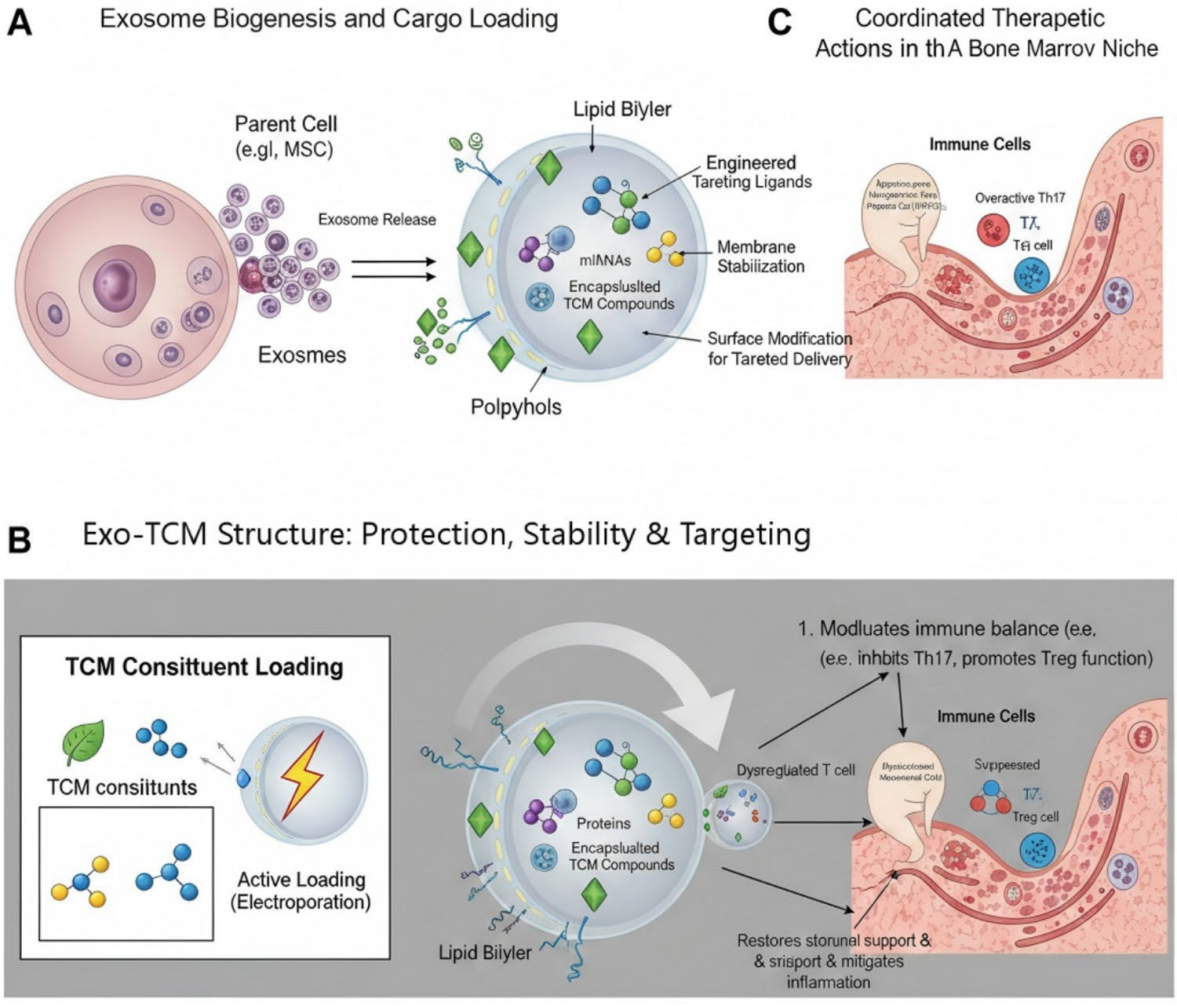
Synergistic mechanisms of the Exo-TCM therapeutic platform. **A** Schematic representation of exosome biogenesis from parental cells and the subsequent encapsulation of Traditional Chinese Medicine (TCM) constituents. **B** The engineered Exo-TCM structure illustrates enhanced stability through membrane interactions, cargo protection within the lipid bilayer lumen, and surface functionalization with targeting ligands. **C** Upon targeted delivery to the aplastic anemia (AA) bone marrow niche, Exo-TCM orchestrates a coordinated therapeutic response by modulating T-cell immunity, repairing the mesenchymal stromal cell (MSC) microenvironment, and promoting hematopoietic stem/progenitor cell (HSPC) survival and function (created with BioRender.com.)

The integration of TCM constituents with exosomes forms a novel therapeutic entity with emergent properties beyond simple drug delivery. As conceptually summarized in Fig. 3, the synergy encompasses upstream modulation of exosome biogenesis, structural and functional enhancement of the vesicle itself, and ultimately, coordinated multi-target actions within the diseased niche. The loading of TCM constituents can influence the exosome output (Fig. 3A), while the resulting Exo-TCM vesicle benefits from enhanced stability and the possibility of targeted delivery through surface engineering (Fig. 3B). This co-delivery system enables a concerted attack on AA pathophysiology: simultaneously rebalancing adaptive immunity, protecting HSPCs, and repairing the stromal microenvironment (Fig. 3C), which is difficult to achieve with monotherapy.

#### TCM constituents modulate exosome biogenesis and cargo sorting

Emerging evidence suggests that bioactive components from TCM may modulate parental cell states to influence exosome biogenesis, subpopulations, and cargo composition. TCM compounds can regulate cellular redox status, inflammatory responses, and signaling pathways in parental cells, thereby altering exosomal output. For instance, herbal constituents like curcumin, resveratrol, and ginsenosides have demonstrated the ability to modulate ROS levels and miRNA expression patterns, which are critical determinants of exosome packaging and secretion [158]. These modifications can lead to quantitative changes in exosome release, qualitative shifts in vesicle subpopulations, and dynamic rewiring of cargo profiles (e.g., proteins, lipids, and nucleic acids) [159]. Notably, TCM-induced alterations in parental cell physiology may specifically enrich therapeutic molecules (e.g., neuroprotective miRNAs) or deplete pathogenic cargo (e.g., pro-inflammatory cytokines) within exosomes [160]. The bidirectional relationship between TCM components and exosome regulation is exemplified by their capacity to influence host EV biogenesis machinery, secretion kinetics, and cargo sorting mechanisms [159]. This paradigm highlights the potential of TCM to engineer functionally optimized exosomes through upstream modulation of parental cell states, offering novel strategies for targeted therapy and precision medicine [89, 161].

#### Enhancement of exosome stability and bioavailability by TCM constituents

Certain bioactive components of Traditional Chinese Medicine (TCM), such as polysaccharides and polyphenols, exhibit intrinsic antioxidant and membrane-stabilizing properties. When loaded into exosomes, these compounds may further reinforce the exosomal lipid bilayer, enhancing structural integrity and resistance to enzymatic degradation in systemic circulation. Polysaccharides may interact with membrane lipids to form protective coatings, as suggested by studies on PELNs demonstrating colloidal stability and high encapsulation efficiency (> 87%) for hydrophobic compounds [162]. Such modifications align with the broader paradigm of exosome engineering, where natural compounds synergize with vesicular carriers to overcome pharmacokinetic limitations (e.g., rapid clearance, low bioavailability) [163]. Future research should quantify the half-life extension conferred by TCM components and elucidate molecular interactions at the exosome membrane interface.

#### Co-delivery and synergistic action at the target site

The dual functionality arises from exosomes' capacity to enhance solubility and stability of challenging TCM compounds while maintaining their own immunomodulatory properties, such as M2 macrophage polarization via IL-10 pDNA delivery or anti-inflammatory effects through curcumin loading [164, 165]. This targeted co-delivery enables simultaneous modulation of multiple pathways—for instance, combining TCM polysaccharides' immunoregulatory effects with exosome-mediated miRNA transfer to achieve synergistic antitumor responses [166]. The systems' biomimetic properties facilitate biological barrier penetration, particularly crucial for neuronal applications where TCM monomers normally face BBB limitations [167]. Furthermore, TCM components can reciprocally modulate exosome biogenesis and cargo composition, creating a self-amplifying therapeutic cycle [168]. Such multi-modal approaches demonstrate superior efficacy in inflammation regulation, wound healing, and disease-modifying effects compared to single-component therapies, as evidenced by enhanced chondroprotection through MATN3/IL-17A delivery in osteoarthritis models [169].

#### Attenuation of TCM toxicity and improvement of biocompatibility

Exsome exhibit remarkable potential in shielding toxic TCM components like Fuzi-derived alkaloids during targeted delivery. The phospholipid bilayer structure provides a physical barrier that encapsulates these bioactive alkaloids, preventing premature leakage and nonspecific interactions with off-target tissues [170, 171]. This compartmentalization significantly reduces systemic toxicity while maintaining the therapeutic payload's integrity until reaching target cells [172, 173]. Studies demonstrate that exosomal membranes effectively protect labile cargo (e.g., nucleic acids) from degradation [174], suggesting similar protective effects for alkaloids. The bilayer's cholesterol and sphingomyelin-rich domains enhance membrane stability [175], further minimizing unintended drug release during circulation. Notably, exosomes' natural tropism for specific recipient cells [176, 177] allows preferential uptake by diseased tissues, thereby concentrating alkaloid delivery at pathological sites while sparing healthy organs. This dual mechanism—physical shielding by the lipid bilayer and biological targeting—synergistically lowers off-target effects [178, 179], ultimately improving the therapeutic index of potent but toxic TCM alkaloids [161].

Understanding these interactions is crucial for rational Exo-TCM design. Future research should focus on elucidating how specific TCM constituents reprogram parent cells and modify exosomal cargo, which will allow us to harness these synergistic effects deliberately, moving from simple loading to intelligent co-engineering.

The therapeutic promise of Exo-TCM lies not merely in co-delivery but in its potential for multi-targeted synergy within the diseased niche. As illustrated in Fig. 2B, upon systemic administration, Exo-TCM is designed to home to the BM, where it can simultaneously promote HSPC survival, modulate immune cell activity, and support stromal repair.

### Characterization techniques

Characterization of exosomes represents a critical step in understanding their biological functions and therapeutic potential. Recent advances in analytical techniques have enabled comprehensive profiling of exosomal properties, including physical characteristics, molecular composition, and functional attributes. Nanoparticle tracking analysis (NTA) with Zeta View has emerged as a powerful tool for determining exosome size distribution and concentration, while simultaneously measuring zeta potential to assess surface charge stability [180]. Transmission electron microscopy (TEM) provides high-resolution visualization of exosome morphology and membrane integrity, serving as a gold standard for ultrastructural validation. Western blot analysis remains indispensable for detecting exosome-specific protein markers such as CD63 and CD9, which confirm vesicle identity and purity [181]. The molecular characterization of exosomal cargo has benefited from technological innovations in omics approaches. The Agilent Bioanalyzer system facilitates quality control and quantification of exosomal RNA, revealing the nucleic acid composition that mediates intercellular communication1. Droplet digital PCR offers sensitive detection of specific RNA species, while lipidomic profiling through mass spectrometry has uncovered dynamic variations in exosomal membrane composition across different isolation batches31. Proteomic analyses have mapped the heterogeneous protein content that reflects parental cell origin and potential biological functions. For therapeutic applications, advanced characterization must address both intrinsic properties and drug loading efficiency. Chromatographic techniques coupled with mass spectrometry enable precise quantification of encapsulated therapeutic compounds, particularly relevant for TCM constituents whose complex structures require detailed chemical profiling. Emerging microfluidic platforms demonstrate improved enrichment efficiency compared to traditional isolation methods, addressing historical challenges of sample volume requirements and purity [182]. These integrated characterization approaches provide essential quality control parameters for developing exosome-based delivery systems, particularly when combining TCM compounds with exosome technology to overcome limitations of poor stability and unclear mechanisms associated with natural products [183, 184]. The field continues to evolve with multimodal characterization strategies that combine physical, molecular, and functional analyses. Such comprehensive profiling is crucial for establishing standardized protocols in exosome research and accelerating clinical translation, particularly in applications involving TCM-derived exosomes or ELNs from medicinal plants [185]. Future directions will likely focus on real-time monitoring techniques and single-vesicle analysis to further elucidate the heterogeneity of exosome populations and their therapeutic mechanisms.

## Challenges and future perspectives

**Fig. 4:**
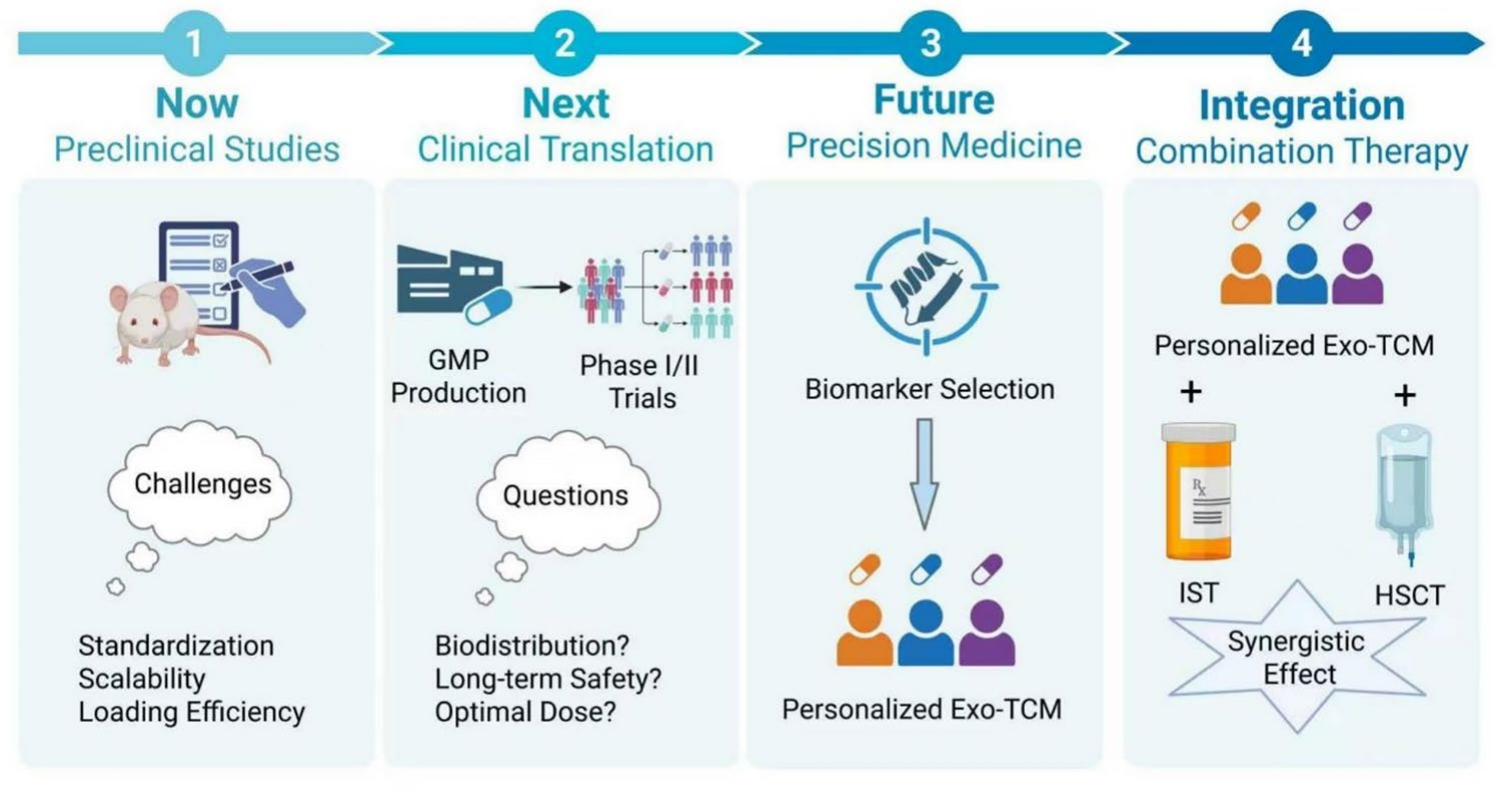
The translational roadmap and future perspectives of Exo-TCM from bench to bedside. This schematic illustrates the proposed pathway for developing Exo-TCM into a viable clinical therapy for AA. The roadmap begins with current preclinical studies (e.g., in vitro and animal models), which face challenges in standardization and scalability. Overcoming these hurdles leads to the stage of clinical translation, encompassing Good Manufacturing Practice production and early-phase trials to evaluate safety, biodistribution, and optimal dosing. Future directions include the development of precision medicine approaches through biomarker-guided patient selection and personalized Exo-TCM formulations. The goal is integration with existing therapies (combination therapy), such as IST or HSCT, to achieve synergistic effects and improve patient outcomes (created with BioRender.com.)

While the preclinical promise of Exo-TCM is compelling, as detailed in previous sections, its path to clinical adoption is fraught with multifaceted challenges. To systematically chart a course for overcoming these hurdles and realizing the full therapeutic potential of this novel strategy, we have formulated a comprehensive translational roadmap (Fig. 4). This visual framework not only delineates the critical path from bench to bedside but also serves to structure our discussion on the necessary future endeavors, which span from addressing immediate technical bottlenecks to envisioning long-term integrative and personalized therapeutic paradigms.

### Current challenges

Despite promising preclinical results, Exo-TCM faces several challenges that hinder their clinical translation (Table 3).

**Table 3:** Comparative analysis of current standard therapies and the novel Exo-TCM strategy for AA

Feature	IST	allo-HSCT	Exo-TCM strategy (Investigational)
Mechanism of action	Broad immunosuppression; eliminates autoreactive T lymphocytes	Replacement of defective HSC pool with healthy donor HSCs	Targeted delivery of TCM constituents to restore immune homeostasis and directly stimulate hematopoiesis & repair BMM
Efficacy & outcomes	▪ Response rate: ~ 60–70%; ▪ CR rate: ~ 10–20%; ▪ Relapse rate: ~ 30–40% at 10 years	▪ Curative potential: High (> 90% survival for MSD); ▪ Graft failure: 5–10%	▪ Preclinical efficacy: Promising in animal models (improved blood counts, BM cellularity); ▪ Multifaceted action: Simultaneously targets HSCs, immunity, and microenvironment
Major risks & limitations	▪ Clonal evolution to MDS/AML (15% at 10 years); ▪ Significant drug toxicity (nephrotoxicity, serum sickness); ▪ High risk of infections	▪ Graft-versus-host disease (GVHD) (acute/chronic) ▪ Transplant-related mortality (TRM) ▪ Donor availability is a major limitation	▪ Long-term safety profile is not yet established ▪ Standardization of exosome production and loading is challenging ▪ Scalability for mass production
Target patient population	First-line for non-transplant candidates, especially older patients	First-line for young, fit patients with a matched sibling donor (MSD)	Potentially all patient groups, particularly ▪ Refractory/relapsed AA; ▪ Patients ineligible for IST or HSCT; ▪ As an adjunct to standard therapy
Key advantages	▪ Avoids transplant-related risks ▪ No donor required	Only curative option with high success rate in ideal candidates	▪ Multitargeted therapy addressing all AA pathogenesis aspects ▪ High biocompatibility and low expected immunogenicity ▪ “Green” strategy leveraging natural products
Refs.	[186]	[187]	[60]

One of the most significant barriers is the lack of standardized protocols for exosome isolation and characterization. Current methods, such as ultracentrifugation, are not only costly but also prone to contamination, leading to variability in exosome quality and purity [188, 189]. This heterogeneity complicates the development of reliable and reproducible Exo-TCM therapies. Additionally, large-scale production of exosomes remains a major hurdle, as existing techniques are not yet optimized for clinical-grade manufacturing [190, 191]. Another critical challenge is the poor drug-loading capacity and targeting efficiency of exosomes. While exosomes exhibit high biocompatibility and low toxicity, their natural cargo may not always align with therapeutic needs, necessitating engineering strategies to improve drug loading and delivery specificity [192, 193]. Furthermore, the complex structures and poor stability of many TCM compounds further complicate their integration into exosome-based delivery systems. Safety and efficacy concern also pose substantial challenges. The long-term biodistribution, potential adverse effects, and immunogenicity of exosomes are not yet fully understood, raising uncertainties about their clinical applicability. Moreover, the lack of personalized approaches in terms of exosome selection, dosage, and administration routes limits their therapeutic precision [194]. Regulatory and translational gaps further hinder progress. Despite extensive preclinical research, there are currently no FDA-approved exosome-based therapies, highlighting the need for more robust clinical trials and regulatory frameworks [195]. The intricate interplay between exosomes and the tumor microenvironment or other disease-specific contexts also requires deeper investigation to optimize therapeutic outcomes [196, 197]. In summary, while Exo-TCM holds immense potential for revolutionizing drug delivery and personalized medicine, overcoming these multifaceted challenges-standardization, scalability, drug loading, safety, and regulatory hurdles—is essential for successful clinical translation. Future research should prioritize elucidating cargo-specific mechanisms, optimize engineering strategies, and advance personalized exosome-based therapies to bridge the gap between bench and bedside.

### Future directions

The integration of exosome research with TCM presents a transformative frontier in precision medicine, yet several critical directions must be prioritized to bridge the gap between molecular insights and clinical applications.

A major bottleneck lies in understanding the functional heterogeneity of exosomes and their cargo-specific roles in TCM-mediated therapies. Future studies should dissect how TCM-derived ELNs regulate immune cells (e.g., CD4 + T cells) to mitigate opportunistic infections or modulate macrophage polarization, aligning with TCM's holistic principles [198, 199]. Advanced multi-omics approaches (e.g., single-vesicle proteomics) could decode the “active components” of TCM-ELNs, accelerating their clinical translation [190].

Standardization of exosome isolation protocols and scalable manufacturing remains a hurdle. Innovations in microfluidics or synthetic biology may enhance yield and purity, particularly for TCM-exosome-like nanovesicles (ELNs), which face challenges in reproducibility due to plant-derived variability. Engineering exosomes with TCM compounds (e.g., Jianpi Ligan Compound [JLC] for liver cancer) could improve targeting and therapeutic consistency [200].

The synergy between TCM’s individualized approach (e.g., constitution-based therapy) and exosome engineering offers promise. Future work should focus on loading exosomes with TCM-active molecules (e.g., monomers or compound prescriptions) to target specific diseases, such as brain disorders or ovarian cancer, while leveraging artificial intelligence (AI) for predictive design [200, 201].

Hydrogel encapsulation, as demonstrated in cancer therapy, significantly enhances the stability, retention, and controlled release of MSC-derived extracellular vehicles (EVs) [202]. This biomaterial strategy offers a promising platform to improve the delivery of TCM-loaded exosomes for AA treatment. By embedding exosomes within hydrogels (e.g., hyaluronic acid or chitosan), sustained and localized release can be achieved in the BM niche. This approach may overcome rapid clearance and enhance immunomodulatory and hematopoietic-regenerative effects. Incorporating targeting ligands further improves precision. Thus, integrating hydrogel technology with TCM-exosome therapeutics represents an advanced, tunable strategy to potentiate efficacy and clinical translation in AA.

Collaborations between TCM experts, nanotechnologists, and clinicians are vital. For instance, exploring the gut-microbiota-exosome axis may reveal novel TCM mechanisms, while explainable AI could decode complex TCM-exosome interactions in drug discovery [203].

## Limitations

This review has several limitations. First, preclinical evidence of Exo-TCM for AA is mostly derived from in vitro and animal models, lacking clinical data to validate efficacy and safety in humans. Second, exosome production faces standardization challenges, as isolation methods (e.g., ultracentrifugation) yield heterogeneous products with inconsistent purity and drug-loading efficiency. Third, TCM constituents’ complexity (e.g., multi-component interactions) and poor stability hinder precise quantification and standardized loading into exosomes. Fourth, the long-term biodistribution, immunogenicity, and potential off-target effects of Exo-TCM remain understudied. Fifth, translational hurdles persist, including scalable manufacturing of clinical-grade Exo-TCM and unclear regulatory frameworks for exosome-based therapies. Lastly, the synergistic mechanisms between specific TCM constituents and exosomes are not fully elucidated, requiring deeper mechanistic exploration.

## Conclusion

AA presents a formidable clinical challenge due to the considerable limitations of current standard treatments, namely IST and allo-HSCT. Persistent issues such as incomplete response, high relapse rates, clonal evolution, donor scarcity, and severe treatment-related toxicities underscore the urgent need for novel, targeted, and well-tolerated therapeutic strategies. This review has highlighted the promising potential of Exo-TCM as an innovative and multifaceted approach to address these unmet medical needs. By synergistically leveraging the targeted delivery capabilities of exosomes and the immunomodulatory and hematopoietic regenerative properties of TCM compounds, Exo-TCM represents a paradigm shift towards a more precise and holistic treatment modality for AA.

Exosomes, as natural DDS, offer unique advantages (biocompatibility, targeted delivery, cargo versatility, BM-blood barrier penetration) over traditional DDS. TCM constituents, with proven efficacy in regulating hematopoiesis and immunity, are valuable AA therapeutic candidates but face delivery bottlenecks (poor solubility, instability, low bioavailability). Exo-TCM resolve these issues and integrate both merits, forming a synergistic system to restore hematopoiesis, repair BM microenvironment, and rebalance immunity.​

Preclinical studies validate Exo-TCM's potential: in vitro, it enhances HSPC clonogenicity, promotes BM MSC function, and regulates immune polarization; in vivo, it improves AA animal models’ blood counts, BM cellularity, and immune balance, mediated via pathways like PI3K/Akt, MAPK, and NF-*κ*B.​

However, Exo-TCM translation faces challenges (exosome large-scale production, TCM standardization, long-term safety evaluations). Future directions include advancing exosome modification, exploring multi-TCM constituent combinations, and integrating Exo-TCM with gene/immunotherapy. With progress in exosome engineering, TCM modernization, and translational research, Exo-TCM is poised to become a clinically applicable AA therapy, improving patient prognosis and quality of life.

## Data Availability

No datasets were generated or analyzed during the current study.

## Abbreviations

AA: Aplastic anemia
allo-HSCT: Allogeneic hematopoietic stem cell transplantation
BBB: Blood–brain barrier
BM: Bone marrow
BMM: Bone marrow microenvironment
DCs: Dendritic cells
DC-EXOs: DC-derived exosomes
DDS: Drug delivery systems
ELNs: Exosome-like nanovesicles
EPOR: Upregulated erythropoietin receptor
EXD: Er-xian decoction
Exo-TCM: Eexosomes loaded with TCM constituents
HSC: Hmatopoietic stem cell
HSPC: Hematopoietic stem/progenitor cells
IST: Immunosuppressive therapy
MGEG: Modified Guilu Erxian Glue
MM: Multiple myeloma
NTA: Nanoparticle tracking analysis
PELNs: Plant-derived exosome-like nanovesicles
TCM: Traditional Chinese medicine
RGP: Rehmannia glutinosa polysaccharide
SAA: Severe AA
SCC: Sodium copper chlorophyllin
SDF-1: Stromal cell-derived factor-1
SJD: Shisiwei Jianzhong Decoction
TEM: Transmission electron microscopy
